# The *catalase* gene family in cucumber: genome-wide identification and organization

**DOI:** 10.1590/1678-4685-GMB-2015-0192

**Published:** 2016-07-25

**Authors:** Lifang Hu, Yingui Yang, Lunwei Jiang, Shiqiang Liu

**Affiliations:** 1 Key Laboratory of Crop Physiology, Ecology and Genetic Breeding, Ministry of Education, Jiangxi Agricultural University, Nangchang, Jiangxi, China; 2 School of Agriculture, Jiangxi Agricultural University, Nanchang, Jiangxi, China; 3 School of Sciences, Jiangxi Agricultural University, Nanchang, Jiangxi, China

**Keywords:** *Cucumis sativus* L., catalase, phylogenetic analysis, gene family, motif

## Abstract

Catalase (CAT) is a common antioxidant enzyme in almost all living organisms. Currently, detailed reports on cucumber (*Cucumis sativus* L.) *CAT* (*CsCAT*) genes and tissue expression profiling are limited. In the present study, four candidate *CsCAT* genes were identified in cucumber. Phylogenetic analysis indicated that *CsCAT1*-*CsCAT3* are closely related to Arabidopsis *AtCAT1*-*AtCAT3*, but no obvious counterpart was observed for *CsCAT4*. Intron/exon structure analysis revealed that only one of the 15 positions was completely conserved. Motif analysis showed that, unlike the *CAT* genes of other species, none of *CsCAT* genes contained all 10 motifs. Expression data showed that transcripts of all of the *CsCAT* genes, except *CsCAT4,* were detected in five tissues. Moreover, their transcription levels displayed differences under different stress treatments.

## Introduction

Catalase (CAT, EC 1.11.1.6) is a common antioxidant enzyme found in nearly all living organisms. It consists of four ferriprotoporphyrin groups per molecule. The typical catalase reaction involves the decomposition of two molecules of hydrogen peroxide (H_2_O_2_) to water (H_2_O) and oxygen (O_2_) (Scandalios, 1987). Catalase exists preferentially in peroxisomes. It is also detected in cytosol, mitochondria, and chloroplasts (Mullen *et al.*, 1997). Plant catalase is usually encoded by a small gene family. Arabidopsis, tobacco, maize, and pumpkins were each found to contain three members of this family, and two each were identified in cottonseed and *Hordeum vulgare* (Kendall *et al.*, 1983; Smith *et al.*, 1984; Willekens *et al.*, 1995; Frugoli *et al.*, 1996; Guan and Scandalios, 1996; Esaka *et al.*, 1997; Mullen *et al.*, 1997; Iwamoto *et al.*, 2000).

Catalase plays a critical role in plant development, defense, and senescence. Catalase scavenges H_2_O_2_ generated during mitochondrial electron transport, the β-oxidation of fatty acids, and most importantly photorespiratory oxidation (Yang and Poovaiah, 2002). Catalase is required when light-dependent plants produce H_2_O_2_ via photorespiration in the peroxisomes. Mutants that lack catalase activity are inviable under conditions in which photorespiration occurs or would occur. Catalase is essential for breaking down the concomitant generation of H_2_O_2_ produced during the first step of β-oxidation during the germination of oilseed plants. Transgenic tobacco overexpressing the *E. coli katE* gene is tolerant to high irradiance under drought conditions, though wild plants suffer severe photosynthesis-induced damage under the same conditions (Shikanai *et al.*, 1998). Arabidopsis catalase 2 knock-out mutants (*cat2*) contain greater amounts of H_2_O_2_, and this is associated with the spread of necrotic lesions (Queval *et al.*, 2007). In tobacco *CAT1* antisense lines, catalase activity is severely reduced and necrotic lesions develop on some of the lower leaves as the H_2_O_2_ level increases (Takahashi *et al.*, 1997). Exogenous application of sweet potato catalase SPCAT1 fusion protein delays or alleviates ethephon-mediated leaf senescence and H_2_O_2_ elevation, which suggests that *SPCAT1* may play a physiological role in H_2_O_2_ homeostasis in leaves, as indicated by developmental cues and environmental stimuli (Chen *et al.*, 2012).

The transcription level of various plant catalases is regulated both temporally and spatially and responds differentially to developmental and environmental stimuli (Guan and Scandalios, 1996; Zimmermann *et al.*, 2006; Du *et al.*, 2008). Tobacco *CAT1* and *CAT2* mRNA transcripts are detected in non-senescent leaves, but *CAT3* is detected in both non-senescent and senescing leaves (Niewiadomska *et al.*, 2009). Maize *CAT1* and *CAT3* are expressed in kernels throughout their development, but *CAT2* is detected only during the later stages of kernel development (Acevedo and Scandalios, 1990; Acevedo *et al.*, 1991). In Arabidopsis, *CAT1* and *CAT2* are mainly expressed in leaves and siliques, whereas *CAT3* is mainly expressed in stem and root. The expression of *CAT2* and *CAT3* has been found to be controlled by circadian rhythms. *CAT2* has been shown to be activated by cold and drought stresses, but *CAT3* is mainly activated by abscisic acid, oxidative treatments, and senescence (Du *et al.*, 2008; Hackenberg *et al.*, 2013; Li *et al.*, 2013; Zou *et al.*, 2015). In hot pepper, different organ-specific expression patterns for *CaCAT1-CaCAT3* are related to circadian rhythms and stress treatments (Lee and An, 2005). In Scots pine, CAT is involved in embryogenesis and cell death processes (Vuosku *et al.*, 2015).

Cucumbers are an economically and nutritionally important vegetable crop cultivated worldwide. It belongs to the *Cucurbitaceae* family. The *CAT* genes exist as a small family in various plant species, but detailed reports on cucumber *CAT* genes and tissue- and stress-specific expression profiles are limited. The recent sequencing of the whole cucumber genome and deep sequencing have made genome-wide analysis of *CAT* genes in cucumbers possible (Huang *et al.*, 2009). In the present study, a genome-wide gene identification, phylogenetic analysis, and expression analysis of *CAT* genes in cucumber and a comparative analysis of cucumber *CAT* genes with those of Arabidopsis, rice, and poplar were conducted. The present findings may serve as an important reference for functional studies of cucumber *CAT* genes.

## Materials and Methods

### Database search for cucumber *CAT* genes

A cucumber catalase sequence (GenBank number GU248529) was used as a query sequence for TBLASTN searches (Altschul *et al.*, 1990) of *CAT* genes encoded in the cucumber genome. The cucumber genome sequence from the Cucumber Genome Initiative (CuGI) obtained and published by the Institute of Vegetables and Flowers of the Chinese Academy of Agricultural Sciences (IVF-CAAS) was used. Default parameters in the TBLASTN searches were wordsize two and extension 11. Redundant sequences with the same scaffold or chromosome location were removed from the data set.

To further confirm the existence of these hypothetical *CAT* genes, the cDNA sequences were first conceptually translated into amino-acid sequences and then searched for the catalase domain using the Simple Modular Architecture Research Tool (SMART) (Letunic *et al.*, 2004).

### Tree building

Multiple sequence alignments were performed on the CAT protein sequences using Clustal X (http://www.clustal.org/) with the default parameters (Larkin *et al.*, 2007), and the alignments were then manually adjusted. A phylogenetic tree was constructed with the aligned CAT protein sequences using MEGA4 (http://www.megasoftware.net/mega4/mega.html) (Tamura *et al.*, 2007) and the neighbor-joining (NJ) method with the following parameters: Poisson correction, pairwise deletion, and bootstrap (1,000 replicates). The constructed tree file was visualized using TreeView 1.6.6 (http://taxonomy.zoology.gla.ac.uk/rod/treeview.html) (Page, 1996).

### Intron/exon structure, genome distribution, and segmental duplication

The DNA and cDNA sequences corresponding to each predicted gene from the cucumber genome and annotation database CuGI were downloaded, and the intron distribution pattern and splicing phase were then analyzed using the web-based bioinformatics tool Gene Structure Display Server (GSDS; http://gsds.cbi.pku.edu.cn/). To obtain information on the location of cucumber *CAT* gene, a map of the distribution of *CsCAT* genes throughout the cucumber genome was drawn using the MapInspect tool. To detect segmental duplication events, the 100 kb DNA segments flanking each *CsCAT* gene were analyzed. Regions in different linkage groups that contained ≥ 6 homologous pairs with < 25 nonhomologous gene interventions were defined as duplicated segments. A gene pair was considered tandemly duplicated when the genes were separated by < 5 intervening genes and shared ≥ 40% amino acid sequence similarity. BioEdit 5.0.6 (http://www.mbio.ncsu.edu/BioEdit/bioedit.html) (Hall, 1999) was used to analyze the *CsCAT* homologs for similarity on the phylogenetic tree.

### Conserved motif prediction

Cucumber CAT proteins were searched for conserved motifs using the Multiple Em for Motif Elicitation to find similar sequences shared by these genes (MEME; http://meme.nbcr.net/meme/tools/meme) (Bailey and Elkan, 1994).

### Reverse Transcription (RT)-PCR analysis of cucumber *CAT* genes

PCR primers were designed to avoid conserved regions. The primer sequences are shown in detail in Table S1. Seeds of the ‘Chinese long' 9930 inbred line, commonly used in modern cucumber breeding (Huang *et al.*, 2009), were germinated and grown in trays containing soil mixture (peat:sand:pumice, 1:1:1, v/v/v). Plants were adequately watered and grown at day/night temperature cycles of 24/18°C with a 16 h photoperiod. For the salt, abscisic acid (ABA) and H_2_O_2_ treatments, seedlings were grown in liquid Murashige and Skoog (MS) medium containing 200 mM NaCl, 100 μM ABA and 10 mM H_2_O_2_, respectively. For the cold treatment, seedlings in the growth chamber were transferred to 4 °C under light conditions. The drought treated seedlings were desiccated.

After treatment for 0, 1, 3, and 6 h, whole seedlings were frozen in liquid nitrogen. Total RNAs of the roots, stems, leaves, flowers, and fruit at the 20 main-stem nodes stages collected under cold, salt, and drought treatment conditions were isolated using the TRIzol Reagent (Tiangen Biotech Co., Ltd. Beijing, China). DNase-treated RNA samples (0.5 μg) were reverse-transcribed using M-MLV reverse transcriptase (Invitogen). The reverse transcription reactions were performed at 42 °C for 1 h using 2 μM oligo-dT18 primer. Two microliters of the first strand cDNAs were used as templates for PCR amplification with a pair of gene-specific primers (Table S1). Samples were denatured for 5 min at 94 °C and then run for about 30 cycles of 30 s at 94 °C, 30 s at 55 °C and 45 s at 72 °C with a final extension of 5 min at 72 °C. For an accurate comparison and quantification of the transcript levels, the exponential phase of PCR amplification was determined by establishing the number of PCR cycles where the products exhibited an exponential phase: 25 cycles for *actin* PCR products and 30 cycles for CAT PCR products. The PCR products were separated by 1.5% agarose gels containing ethidium bromide and photographed under UV light. The results were confirmed using three independent biological replicates. The cucumber *ACTIN* cDNA fragment (161 bp) was used as an internal standard for normalizing cDNA concentration variations.

## Results

### Identification of four *CsCAT* genes

To identify the full complement of *CAT* genes in cucumber genomes, a cucumber catalase sequence (GenBank accession number GU248529) was used as a BLAST query sequence, and four candidate sequences were identified. The same four sequences were also obtained from Cucumber Genome Initiative (CuGI) using the HMM program based on multiple sequence alignment results of Arabidopsis CAT domain sequences. To further verify the reliability of these candidate sequences, SMART analysis was performed and all four sequences showed a typical CAT domain. The four cucumber *CAT* genes (*CsCAT*; Table 1) were subjected to further analysis. The number designation was based on the order of multiple sequence alignments. For study purposes, each was provisionally distinguished by a generic name, viz., *CsCAT1*-*CsCAT4*.

**Table 1:** The *CsCAT* genes identified in this study. The number designations are based on the order of the multiple sequence alignments.

Serial No.	Gene Name	Gene ID	Chromosamal location	CuGI(5'-3')	Length (a.a.)	Group name
1	*CsCAT1*	Csa014901	4	14579559-14582377	536	III
2	*CsCAT2*	Csa014900	4	14584724-14588175	458	I
3	*CsCAT3*	Csa013194	6	14161472-14164768	483	II
4	*CsCAT4*	Csa022770	Scaffold000416	9221-11455	744	II

### Phylogenetic analysis of the *CsCAT* genes

**Figure 1:**
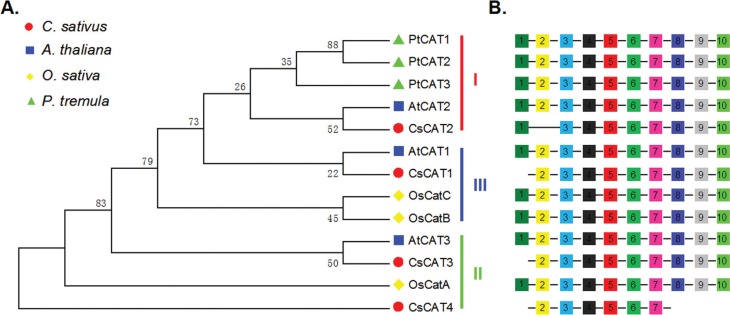
Plant catalase genes. (A) Neighbor-joining tree for CAT sequences of cucumber and other species. (B) Distribution of conserved motifs in four cucumber CsCATs, three rice OsCATs, three Arabidopsis AtCATs, and three poplar PtCAT proteins identified using the MEME search tool. Each motif is represented by a colored box and a number. The order of the motifs corresponds to the position of motifs in individual protein sequences. Details regarding the motifs are given in Table S3.

To investigate the evolutionary relationships of cucumber *CsCATs* to those in other species, an unrooted phylogenetic tree using bootstrap analysis (1,000 replicates) was built from alignments of the complete protein sequences of four cucumber *CsCATs*, three rice *OsCATs*, three Arabidopsis *AtCATs*, and three poplar *PtCATs* (Figure 1A). The results showed that, like the findings observed in other species (Mhamdi *et al.*, 2010a), these 13 *CATs* could be divided into three classes (I–III), containing five, four, and four genes, respectively (Figure 1A). All three poplar *CAT* genes (*PtCAT1*-*PtCAT3*) existed in the form of an exclusive cluster belonging to class I. Among the four cucumber *CsCATs*, *CsCAT1* was grouped in class III, *CsCAT2* in class I, and *CsCAT3* and *CsCAT4* in class II. Compared to genes from rice and poplar, it seemed that *CsCAT1*, *CsCAT2* and *CsCAT3* were more related to *AtCAT1*, *AtCAT2*, and *AtCAT3* in Arabidopsis, respectively. *CsCAT4* was at the basal position of the tree and far distant from the other clades.

### Sequence analysis and multiple sequence alignment

The open reading frame length of the four *CsCAT* genes varied from 1377 (*CsCAT2*) to 2235 bp (*CsCAT4*), encoding polypeptides of 458–744 amino acids, with a predicted molecular weight range of 53.49–82.90 kDa. Multiple alignment demonstrated that all CsCAT proteins contained a highly conserved region of about 380 amino acid residues in their N-terminal portion and 60 amino acid residues in their C-terminal portion corresponding to the catalase and catalase-rel domains, respectively. The homologies ranged from 40% to 76%, with an average homology of only 57.33%, suggesting a high sequence diversity between cucumber *CsCAT* genes. Detailed information is given in Table S2. Multiple sequence alignment showed three conserved catalytic amino acid residues for CAT enzyme (His-71, Asn-144, and Tyr-354 in the crab sequence) to be completely conserved in all *CsCATs* except *CsCAT2* (Figure S1). The catalase proximal active site signature FDRERIPERVVHAKGAGA (residues 60–77), was also found to be highly conserved. Another sequence, RLFSYNDTH (residues 350–358), representing the proximal heme-ligand signature was only conserved in *CsCAT1*-*CsCAT3* (Figure S1).

The plant CAT enzyme activity is known to be mainly localized in the peroxisome, which was targeted by the particular peroxisomal targeting signal (PTS). The consensus amino acid sequence for putative peroxisomal targeting, Ser/Glu/Cys-Lys/Arg/His-Leu, was located at nine amino acids from the carboxy terminus (Subramani, 1998). The current alignment analysis results showed that the classical peroxisomal targeting sequence was only observable at the C-terminus of *CsCAT2*. Another internal consensus tripeptide PTS1-like motif (QKL/I/V) was reported by Kamigaki (Kamigaki *et al.*, 2003). It is located upstream of the Ser/Glu/Cys-Lys/Arg/His-Leu motif and was also found at the same sites as *CsCAT2* and *CsCAT3* (Figure S1). The PTS1-like motif sequence can be attributed to the efficient importation of catalase into peroxisomes.

### Structure and evolution of the *CsCAT* genes

**Figure 2:**
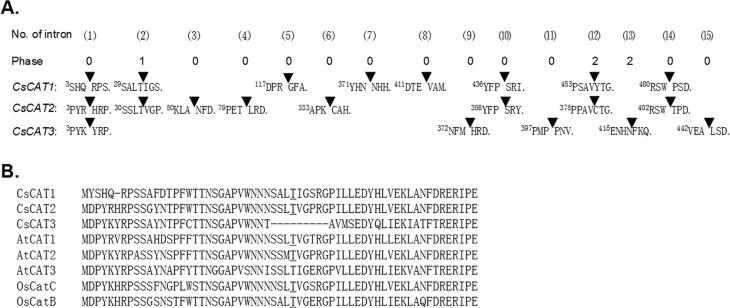
Sequence analysis of plant catalase genes. (A) Nucleotide sequences surrounding splice sites in three cucumber *CsCAT* genes and the consensus sequence for the proto-splice site. Amino acid residues and codons (*solid triangles*) that are split by introns are also shown. (B) Amino acid sequences flanking the intron splice sites (*underlined*) in three cucumber CsCATs, two rice OsCATs, and three Arabidopsis AtCATs are shown. The amino acid Thr that is split by intron, which is widely conserved in the CAT family, is underlined.

The pattern of intron positioning can provide some clues regarding evolutionary history. To investigate intron number and positions, a comparison of the full-length cDNA sequences with the corresponding genomic DNA sequences was performed. A previous study revealed that the rice and Arabidopsis *CAT* genes contained six to seven introns (Mhamdi *et al.*, 2010b). A similar situation was observed in cucumber *CsCAT1–CsCAT3*, which had five to eight introns. *CsCAT4* was an exception to this rule, showing no introns (Figure 2A). Further analysis showed a total of 15 intron positions in *CsCAT1*–*CsCAT3*. Among them, only one position (1) was completely conserved in three genes (indicated with a solid triangle). Of the remaining 14 positions, four positions (2, 10, 12, 14) were highly conserved between *CsCAT1* and *CsCAT2*. However, no position was conserved between *CsCAT1* and *CsCAT3* or between *CsCAT2* and *CsCAT3*. The intron phase analysis demonstrated that all the positions showed phase 0 (splicing occurring after the third nucleotide of the codon), except positions 12 and 13 with phase 2 (splicing occurring after the second nucleotide of the codon) and position two with phase 1.

In Arabidopsis, the *CAT* genes had one intron that splits the Thr in the consensus sequence of SS(M/L)T(V/I)G(P/T/E)RGP (Frugoli *et al.*, 1996). The current analysis showed that *CsCAT1* and *CsCAT2* contained one intron at the conserved Thr site, as expected, but no such situation was observed in *CsCAT3* (Figure 2B).

### *CsCAT* conserved motif prediction

To investigate the characterized regions of cucumber CAT proteins, the online MEME motif search tool was used to analyze the distribution of the motifs in *CsCATs* in rice, Arabidopsis, and poplar. A total of 10 motifs, here called motifs 1–10, were identified (Figure 1B; Table S3). Among them, motifs 1–9 represented the catalase domain and motif 10 the catalase-rel region. It is worthy to note that the 10 motifs were all observed in rice, Arabidopsis, and poplar *CATs*, but no *CsCAT* gene contained all 10 motifs. For example, motif 1 was absent from *CsCAT1*, *CsCAT3*, and *CsCAT4*, motif 2 was absent from *CsCAT2*, and motifs 8–10 were absent from *CsCAT4*.

### Detection of 4 *CsCAT* genes in different tissues and stress conditions

**Figure 3:**
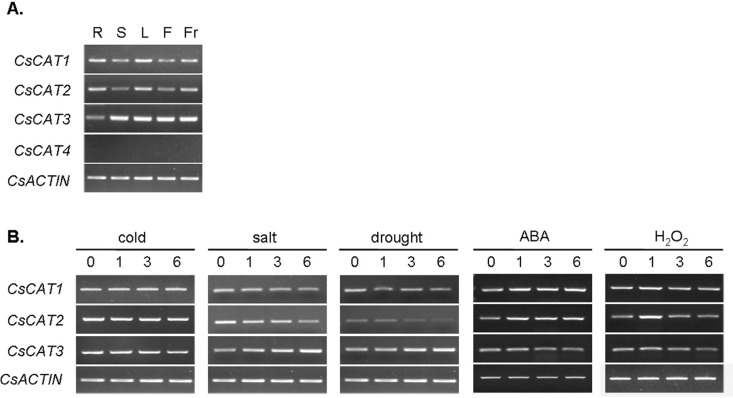
RT-PCR analysis of cucumber *CsCAT* genes, (A) in different tissues and, (B) under salt, cold, drought, H_2_O_2-_, and ABA stress conditions for 0, 1, 3, and 6 h. RT-PCR was performed using primers specific to the *CsCAT* genes. PCR products were run on 1.5% agarose gels. *CsACTIN* primers that generated a 161 bp product, which was used as the internal standards for each *CsCAT* gene. Sources of the samples are as follows: (R) root, (S) stem, (L) leaves, (F) flowers, and (Fr) fruit.

RT-PCR analysis was performed on RNA from root, stem, leaves, flowers, and fruit to assess the expression patterns of the four *CsCAT* genes (Figure 3A). The RT-PCR results showed that *CsCAT1–CsCAT3* are expressed in all tissues investigated, but no *CsCAT4* expression was detected in any of these. *CsCAT1* had expression patterns similar to *CsCAT2*, with a higher transcript signal in root, leaves, and fruit. *CsCAT3* displayed noticeably high expression signals in stem, leaves, flower, and fruit. Catalase is known to play a vital role in response to a subset of biotic and abiotic stress reactions.

To investigate the response of *CsCAT* genes to various abiotic stresses, RT-PCR was performed on 7-day-old seedlings treated under five different sets of abiotic stress conditions (salt, cold, drought, H_2_O_2_, and ABA) (Figure 3B). The data indicated that *CsCAT1–CsCAT3* expression levels were reduced or increased relative to controls in at least one of the stress conditions examined. The level of transcription of *CsCAT1* was increased in ABA treatment and that of *CsCAT2* was enhanced by ABA and in the sample 1 for H_2_O_2_, but levels of *CsCAT1* and *CsCAT2* were moderately diminished for salt and drought. *CsCAT3* transcript levels were slightly increased under salt, drought, and ABA stress conditions. Its transcription levels were enhanced under salt and drought conditions but reduced under ABA stress conditions.

## Discussion

Generally, plant catalases comprise a small gene family only. There are three catalases each in *Arabidopsis thaliana*, *Nicotiana tabacum*, and *Zea mays* and two each in *Hordeum vulgare* and cottonseed (Kendall *et al.*, 1983; Smith *et al.*, 1984; Willekens *et al.*, 1995; Frugoli *et al.*, 1996; Guan and Scandalios, 1996; Esaka *et al.*, 1997; Mullen *et al.*, 1997; Iwamoto *et al.*, 2000). The current study showed that cucumber contains four *CAT* genes (*CsCAT1*-*CsCAT4*), i.e. at least one more than those of the above mentioned species. The genomic distribution analysis showed that *CsCAT1*–*CsCAT3* are located on chromosome scaffolds, whereas *CsCAT4* was found on an unassembled sequence, scaffold000416. Hence, if *CsCAT4* is removed from the dataset, the number of *CAT* genes in cucumber genome would drop to three, which makes it comparable to those in other reported species.

Sequence analysis showed that the catalytic amino acids, proximal heme-ligand signature sequence and catalase proximal active site signature were highly conserved among *CsCAT1*–*CsCAT3*, suggesting that these genes are likely to have functions similar to those of catalases in other species. The predicted peroxisome targeting signal analysis showed that *CsCAT2* and *CsCAT3* contain the putative peroxisomal targeting signals S/E/C-K/R/H-L, QKL/I/V, or both, indicating that *CsCAT2* and *CsCAT3* could be peroxisomal catalases. Nonetheless, further experimental analysis is needed for confirmation.

The phylogenetic tree analysis indicated that cucumber *CsCAT1*, *CsCAT2*, and *CsCAT3* are more closely related to *AtCAT1*, *AtCAT2*, and *AtCAT3* in Arabidopsis, respectively, than to other counterparts in other species. *AtCAT1* and *AtCAT2* were found to be mainly expressed in leaves and siliques, but *AtCAT3* was preferentially expressed in stems and roots (Du *et al.*, 2008).

The current expression analysis showed that *CsCAT1* and *CsCAT2* are represented by transcripts in photosynthetic tissue, such as leaves, but also in root and fruit. *CsCAT3* showed transcripts in flowers and fruit, and also in stem and leaves. Based on the expression pattern comparison, *CsCATs* exhibited an expression pattern similar to their Arabidopsis counterparts. This suggests that the three *CsCAT* genes might play similar roles in cucumber development.

Under different stress treatment conditions, *CsCAT1* was only activated by abscisic acid treatment, whereas the abundance of its Arabidopsis counterpart, the *CAT1* transcript, has been shown significantly increased by cold, drought, abscisic acid, and oxidative treatments (Du *et al.*, 2008). *CsCAT2* transcription was reduced in response to drought, whereas Arabidopsis *CAT2* transcription was elevated (Du *et al.*, 2008). Similarly, *CsCAT3* transcription was enhanced under salt and drought stress conditions and reduced under ABA stress conditions, while Arabidopsis *CAT3* was increased by abscisic acid and oxidative treatments (Du *et al.*, 2008). Based on the analysis of different environment stimuli, obvious differences were observed between cucumber *CsCAT* and their Arabidopsis counterparts. This leads to conclude that the activation of cucumber *CsCATs* may differ in response to different abiotic and biotic stresses.

Intron analysis demonstrated that *CsCAT1* and *CsCAT2* each contained one conserved intron and Thr in the consensus sequence of SS(M/L)T(V/I)G(P/T/E)RGP as expected, but this was not observed in *CsCAT3*, suggesting that *CsCAT3* might have evolved more recently. Motif analysis showed that none of the *CsCAT* genes contained all of the expected motifs. This makes them different from the *CAT* genes in other species, and suggests that some motifs might have been lost from cucumber after the divergence of monocots and dicots, or may have evolved solely in cucumbers after the divergence.

In summary, we performed extensive analyses of the four cucumber *CAT* genes and compared them to three rice *OsCATs*, three Arabidopsis *AtCATs*, and three poplar *PtCATs*. The 13 *CAT* genes clustered into three classes (I–III), which were in general agreement with reported results (Mhamdi *et al.*, 2010a). The protein sequence analysis showed that *CsCAT2* and *CsCAT3* might be peroxisomal catalases. The expression analysis of the four cucumber *CsCAT* genes in root, stem, leaves, flowers, and fruit showed that all genes were expressed in at least one tissue. Furthermore, their expression patterns displayed differences when exposed to stress conditions (cold, salt, drought, H_2_O_2_, and ABA treatment). The comprehensive data collected here may be useful for future analysis of the biological functions of *CAT* family genes in cucumber growth, development, and responses to different stresses.

## Supplementary material

The following online material is available for this article:

Figure S1 - Multiple alignment of the deduced catalase amino acid sequences.

Table S1 - Primers used in RT-PCR.

Table S2 - Sequence alignment between cucumber four *CsCAT* genes.

Table S3 - Summary of the conserved motifs (CMs) within the CsCAT family.
