# Supplementary material for: The catalase gene family in cucumber: genome-wide identification and organization
Source: Genet Mol Biol. 2016 Jul 25;39(3):408–15. doi: 10.1590/1678-4685-GMB-2015-0192 (PMC5004828; doi:10.1590/1678-4685-GMB-2015-0192)
Supplement: Supplementary file 1 [file 1415-4757-gmb-1678-4685-GMB-2015-0192-Suppl02.pdf]

Table S1. Primers used in RT-PCR.

Primer name	Sequence (5' to 3')
CsCAT1_F	CCGAGAGGTATCCTCAACCA
CsCAT1_R	TCGTTCTTGCCTGTCTGATG
CsCAT2_F	ACAATCACCACGAGGGTTTC
CsCAT2_R	GACAAAGCATCCACCCATCT
CsCAT3_F	ATGCTCCCAAATGTCCTCAC
CsCAT3_R	GCCCATGATCGGTATCTGTC
CsCAT4_F	ACGGTTGATGCGGTGATTAT
CsCAT4_R	AATGCCTTCTTCCCCTTGAT
CsACTIN_F	GACATTCAATGTGCCTGCTATG
CsACTIN_R	CATACCGATGAGAGATGGCTG
