# Supplementary material for: The catalase gene family in cucumber: genome-wide identification and organization
Source: Genet Mol Biol. 2016 Jul 25;39(3):408–15. doi: 10.1590/1678-4685-GMB-2015-0192 (PMC5004828; doi:10.1590/1678-4685-GMB-2015-0192)
Supplement: Supplementary file 2 [file 1415-4757-gmb-1678-4685-GMB-2015-0192-Suppl04.pdf]

Table S3. Summary of conserved motifs(CMs) within the CsCAT family

CM-1

Name	Start	p-value		Sites	
PtCAT3	1	3.83E-51		MDPYKYRPSSSYNSPFFTTNSGAPVWNNNSSLTVGSRGPIL	LEDYHLVEKL
PtCAT2	1	1.20E-48		MDPYKHRPSSAFNSPYWTTNSGASVWNNNSSLTVGSRGPIL	LEDYHLVEKI
CsCAT2	1	1.35E-48		MDPYRHRPSSGYNTPFWTTNSGAPVWNNNSSLTVGPRGPIL	LEDYHLVEKL
AtCAT2	1	1.51E-48		MDPYKYRPASSYNSPFFTTNSGAPVWNNNSSMTVGPRGPIL	LEDYHLVEKL
PtCAT1	1	1.51E-48		MDPYKYRPSSAFGTPYWTTNSGAPVWNNNSSLTVGSRGPIL	LEDYHLVEKL
OsCatB	1	4.95E-47		MDPYKHRPSSGSNSTFWTTNSGAPVWNNNSALTVGERGPIL	LEDYHLIEKL
OsCatC	1	2.23E-45		MDPYKHRPSSSFNGPLWSTNSGAPVWNNNNSLTVGSRGPIL	LEDYHLVEKL
AtCAT1	1	5.28E-45		MDPYRVRPSSAHDSPFFTTNSGAPVWNNNSSLTVGTRGPIL	LEDYHLLEKL
AtCAT3	1	4.02E-41		MDPYKYRPSSAYNAPFYTTNGGAPVSNNISSLTIGERGPVL	LEDYHLIEKV
OsCatA	1	3.39E-39		MDPCKFRPSSSFDTKTTTTNAGAPVWNDNEALTVGPRGPIL	LEDYHLIEKV

CM-2

Name	Start	p-value		Sites	
AtCAT2	42	1.96E-62	MTVGPRGPIL	LEDYHLVEKLANFDRERIPERVVHARGASAKGFFEVTHDISNLTCADFLR	APGVQTPVIV
OsCatC	42	8.28E-62	LTVGSRGPIL	LEDYHLVEKLANFDRERIPERVVHARGASAKGFFEVTHDITHLTCADFLR	APGVQTPVIV
CsCAT1	41	8.28E-62	LTIGSRGPIL	LEDYHLVEKLANFDRERIPERVVHARGASAKGFFEVTHDITHLTCADFLR	APGTQTPVIV
PtCAT1	42	1.53E-61	LTVGSRGPIL	LEDYHLVEKLANFDRERIPERVVHARGASAKGFFEVTHDISGLTCADFLR	APGVQTPVIV
PtCAT3	42	9.60E-61	LTVGSRGPIL	LEDYHLVEKLANFDRERIPERVVHARGASAKGFFEVTNDISHLTCADFLR	APGVQTPVIV
OsCatB	42	1.25E-60	LTVGERGPIL	LEDYHLIEKLAQFDRERIPERVVHARGASAKGFFEVTHDISHLTCADFLR	APGVQTPVIV
PtCAT2	42	1.94E-60	LTVGSRGPIL	LEDYHLVEKIANFDRERIPERVVHARGASAKGFFEVTHDISNLSCADFLR	APGVQTPVIV
AtCAT3	42	1.99E-58	LTIGERGPVL	LEDYHLIEKVANFTRERIPERVVHARGISAKGFFEVTHDISNLTCADFLR	APGVQTPVIV
AtCAT1	42	1.55E-56	LTVGTRGPIL	LEDYHLLEKLANFDRERIPERVVHARGASAKGFFEVTHDITQLTSADFLR	GPGVQTPVIV
OsCatA	42	1.09E-54	LTVGPRGPIL	LEDYHLIEKVAHFARERIPERVVHARGASAKGFFECTHDVTDITCADFLR	SPGAQTPVIV
CsCAT3	33	1.09E-54	APVWNNTAVM	SEDYQLIEKIATFTRERIPERVVHARGASAKGFFEVTHDVSDLTCADFLR	APGVQTPVIV
CsCAT4	101	9.26E-35	LRAGTRGPTL	LEDFILREKITHFDHERIPERIVHARGSAAHGYFQPYKSLKEITKADFLS	DPNKITPVFV

CM-3

Name	Start	p-value		Sites	
AtCAT2	96	2.82E-62	CADFLRAPGV	QTPVIVRFSTVIHERGSPETLRDPRGFAVKFYTREGNFDLVGNNFPVFFI	RDGMKFPDMV
PtCAT3	96	2.82E-62	CADFLRAPGV	QTPVIVRFSTVIHERGSPETLRDPRGFAVKFYTREGNFDLVGNNFPVFFI	RDGMKFPDMV
PtCAT1	96	2.82E-62	CADFLRAPGV	QTPVIVRFSTVIHERGSPETLRDPRGFAVKFYTREGNFDLVGNNFPVFFI	RDGMKFPDMV
CsCAT1	95	6.97E-62	CADFLRAPGT	QTPVIVRFSTVIHERGSPETLRDPRGFAVKFYTREGNFDLVGNNFPVFFV	RDGMKFPDMV
AtCAT1	96	6.97E-62	SADFLRGPGV	QTPVIVRFSTVIHERGSPETLRDPRGFAVKFYTREGNFDLVGNNFPVFFV	RDGMKFPDMV
PtCAT2	96	6.97E-62	CADFLRAPGV	QTPVIVRFSTVIHERGSPETLRDPRGFAVKFYTREGNFDLVGNNFPVFFV	RDGMKFPDMV
OsCatC	96	8.47E-61	CADFLRAPGV	QTPVIVRFSTVIHERGSPETLRDPRGFAIKFYTREGNWDLVGNNFPVFFI	RDGMKFPDMV
CsCAT3	87	9.67E-61	CADFLRAPGV	QTPVIVRFSTVIHERGSPETLRDPRGFAVKFYTREGNFDIVGNNFPVFFV	RDAMQFPDVI
OsCatB	96	1.99E-60	CADFLRAPGV	QTPVIVRFSTVVHERGSPETLRDPRGFAVKFYTREGNFDLVGNNMPVFFI	RDGMKFPDMV
CsCAT2	62	3.71E-60	ANFDRERIPE	RTPVIVRFSTVIHERGSPETLRDPRGFAVKFYTREGNFDLVGNNFPVFFI	RDGMKFPDMV
OsCatA	96	3.61E-59	CADFLRSPGA	QTPVIVRFSTVIHERGSPETIRDPRGFAVKFYTREGNWDLLGNNFPVFFI	RDGIKFPDVI
AtCAT3	96	6.82E-57	CADFLRAPGV	QTPVIVRFSTVVHERASPETMRDIRGFAVKFYTREGNFDLVGNNTPVFFI	RDGIQFPDVV

CsCAT4	155	1.21E-42	**KADFLSDPNK**	**ITPVFVRFSTVQGGAGSADTVRDIRGFATKFYTEEGIFDLVGNNTPVFFI**	**QDAHKFPDFV**

CM-4

Name	Start	p-value		Sites	
CsCAT1	145	4.56E-38	**VGNNFPVFFV**	**RDGMKFPDMVHALKPNPKSHIQENWRILD**	**FFSHHPESLH**
AtCAT1	146	4.56E-38	**VGNNFPVFFV**	**RDGMKFPDMVHALKPNPKSHIQENWRILD**	**FFSHHPESLH**
CsCAT2	112	4.56E-38	**VGNNFPVFFI**	**RDGMKFPDMVHALKPNPKSHIQENWRILD**	**FFSHHPESLN**
AtCAT2	146	4.56E-38	**VGNNFPVFFI**	**RDGMKFPDMVHALKPNPKSHIQENWRILD**	**FFSHHPESLN**
PtCAT3	146	4.56E-38	**VGNNFPVFFI**	**RDGMKFPDMVHALKPNPKSHIQENWRILD**	**FFSHHPESLH**
PtCAT2	146	4.56E-38	**VGNNFPVFFV**	**RDGMKFPDMVHALKPNPKSHIQENWRILD**	**FFSHHPESLH**
PtCAT1	146	4.56E-38	**VGNNFPVFFI**	**RDGMKFPDMVHALKPNPKSHIQENWRILD**	**FFSHHPESLH**
OsCatC	146	5.59E-36	**VGNNFPVFFI**	**RDGMKFPDMVHSLKPNPKSHVQENWRILD**	**FFSHHPESLH**
OsCatB	146	2.04E-31	**VGNNMPVFFI**	**RDGMKFPDMVHAFKPSPKTNMQENWRIVD**	**FFSHHPESLH**
AtCAT3	146	4.59E-31	**VGNNTPVFFI**	**RDGIQFPDVVHALKPNPKTNIQEYWRILD**	**YMSHLPESLL**
CsCAT3	137	3.47E-30	**VGNNFPVFFV**	**RDAMQFPDVIRAFKPNPKSHIQEPWRILD**	**FCSYHPESLL**
OsCatA	146	4.32E-28	**LGNNFPVFFI**	**RDGIKFPDVIHAFKPNPRSHVQEYWRVFD**	**FLSHHPESLH**
CsCAT4	205	1.71E-16	**VGNNTPVFFI**	**QDAHKFPDFVHAVKPEPHWAIPQGQSAHD**	**TFWDYVSLQP**

CM-5

Name	Start	p-value		Sites	
PtCAT1	185	8.35E-64	**FFSHHPESLH**	**MFSFLFDDLGVPQDYRHMEGSGVNTYMLINKAGKAHYVKFHWKPTCGVKC**	**LLEDEAVKVG**
AtCAT2	185	1.53E-63	**FFSHHPESLN**	**MFTFLFDDIGIPQDYRHMDGSGVNTYMLINKAGKAHYVKFHWKPTCGVKS**	**LLEEDAIRVG**
AtCAT1	185	7.97E-63	**FFSHHPESLH**	**MFSFLFDDLGIPQDYRHMEGAGVNTYMLINKAGKAHYVKFHWKPTCGIKC**	**LSDEEAIRVG**
CsCAT2	151	7.60E-62	**FFSHHPESLN**	**MFTFLFDDIGIPQDYRHMDGSGVNTYTLINKAGKAHYVKFHWRPTCGVKS**	**LLEEDAIRVG**
PtCAT2	185	1.14E-61	**FFSHHPESLH**	**MFSFLFDDLGVPQDYRHMEGAGVNTYTLINKAGKANYVKFHWKPTCGVKC**	**LLEDEAIKVG**
PtCAT3	185	2.55E-60	**FFSHHPESLH**	**MFSFLLDDIGVPQDYRHMEGSGVNTYTLINKAGKAYYVKFHWKPTCGVKS**	**LLEDEAIKVG**
CsCAT1	184	1.07E-58	**FFSHHPESLH**	**MFTFLFDDLGIPQDYRHMDGSGVNTYTLINKEGKVHYVKFHWRPTCGVKT**	**LLDEVAIRVG**
OsCatC	185	2.85E-57	**FFSHHPESLH**	**MFTFLFDDIGIPADYRHMDGSGVNTYTLVNRAGKSHYVKFHWKPTCGVKS**	**LLDDEAVTVG**
OsCatB	185	6.15E-57	**FFSHHPESLH**	**MFSFLFDDVGIPLNYRHMEGFGVNTYTLINKDGKPHLVKFHWKPTCGVKC**	**LLDDEAVTVG**
AtCAT3	185	6.79E-50	**YMSHLPESLL**	**TWCWMFDDVGIPQDYRHMEGFGVHTYTLIAKSGKVLFVKFHWKPTCGIKN**	**LTDEEAKVVG**
OsCatA	185	3.60E-48	**FLSHHPESLH**	**TFFFLFDDVGIPTDYRHMDGFGVNTYTFVTRDAKARYVKFHWKPTCGVSC**	**LMDDEATLVG**
CsCAT3	176	7.81E-48	**FCSYHPESLL**	**SFAWFYDDVGIPINYRHMEGFGVQAYSLINKSGKARLVKFHWKPTCGVKS**	**MLEEEAIRIG**
CsCAT4	248	3.38E-34	**YVSLQPETLH**	**NVMWAMSDRGIPRSYRTMEGFGIHTFRLINAEGKATFVRFHWKPVAGKAS**	**LVWDEAQKLT**

CM-6

Name	Start	p-value		Sites	
PtCAT2	239	6.44E-52	**TCGVKCLLED**	**EAIKVGGANHSHATQDLYDSIAAGNYPEWKLFIQTIDPDHE**	**DSFDFDPLDV**
CsCAT2	205	3.08E-50	**TCGVKSLLEE**	**DAIRVGGSNHSHATQDLYDSIAAGNYPEWKLFIQTIDPDHE**	**DRYDFDPLDV**
OsCatC	239	5.80E-50	**TCGVKSLLDD**	**EAVTVGGTNHSHATQDLYDSIAAGNFPEWKLFIQTIDPDHE**	**DRFDFDPLDV**
PtCAT1	239	3.56E-49	**TCGVKCLLED**	**EAVKVGGTNHSHATQDLYDSIAAGTYPEWKLFIQTIDPDHE**	**ARFDFDPLDV**
AtCAT2	239	5.11E-49	**TCGVKSLLEE**	**DAIRVGGTNHSHATQDLYDSIAAGNYPEWKLFIQIIDPADE**	**DKFDFDPLDV**
PtCAT3	239	2.30E-48	**TCGVKSLLED**	**EAIKVGGSNHSHATQDLYDSIKAGNYPEWKLFIQIIDPADE**	**DKFDFDPLDV**
CsCAT1	238	3.19E-47	**TCGVKTLLDE**	**VAIRVGGSNHSHATQDLYDSIAAGNYPEWELYIQTIDPDHE**	**DKYDFDPLDV**
OsCatB	239	9.07E-47	**TCGVKCLLDD**	**EAVTVGGTCHSHATKDLTDSIAAGNYPEWKLYIQTIDPDHE**	**DRFDFDPLDV**
OsCatA	239	3.52E-44	**TCGVSCLMDD**	**EATLVGGKNHSHATQDLYDSIAAGNFPEWKLFVQVIDPEEE**	**ERFDFDPLDD**

AtCAT1	239	7.36E-44	**TCGIKCLSDE**	**EAIRVGGANHSHATKDLYDSIAAGNYPQWNLFVQVMDPAHE**	**DKFDFDPLDV**
CsCAT3	230	4.49E-43	**TCGVKSMLEE**	**EAIRIGGTNHSHATQDLYESIAAGNFPEWRLYIQTIDYDDQ**	**NNFDFEPLDT**
AtCAT3	239	5.37E-43	**TCGIKNLTDE**	**EAKVVGGANHSHATKDLHDAIASGNYPEWKLFIQTMDPADE**	**DKFDFDPLDV**

CM-7

Name	Start	p-value		Sites	
CsCAT2	249	1.56E-57	**TIDPDHEDRY**	**DFDPLDVTKTWPEDILPLQPVGRMVLNKNIDNFFAENEQLAFCPA**	**IIVPGIYYSD**
AtCAT2	283	1.56E-57	**IIDPADEDKF**	**DFDPLDVTKTWPEDILPLQPVGRMVLNKNIDNFFAENEQLAFCPA**	**IIVPGIHYSD**
CsCAT1	282	8.79E-57	**TIDPDHEDKY**	**DFDPLDVTKTWPEDILPLQPVGRLVLNKNIDNFFAENEQLAFCPA**	**IIVPGIYYSD**
PtCAT1	283	8.79E-57	**TIDPDHEARF**	**DFDPLDVTKTWPEDILPLQPVGRLVLNKNIDNFFAENEQLAFCPA**	**IVVPGVYYSD**
OsCatB	283	1.14E-56	**TIDPDHEDRF**	**DFDPLDVTKTWPEDIIPLQPVGRMVLNKNIDNFFAENEQLAFCPA**	**IIVPGIHYSD**
PtCAT2	283	5.86E-56	**TIDPDHEDSF**	**DFDPLDVTKIWPEDILPLQPVGRLVLNKNIDNFFAENEQLAFCPA**	**IVVPGIYYSD**
PtCAT3	283	7.61E-55	**IIDPADEDKF**	**DFDPLDVTKTWPEDILPLMPVGRLVLNKNIDNFFAENEQLAFCPA**	**IIVPGIYYSD**
OsCatC	283	5.81E-54	**TIDPDHEDRF**	**DFDPLDVTKTWPEDIVPLQPVGRMVLNRNIDNFFSENEQLAFCPG**	**IIVPGIYYSD**
AtCAT1	283	7.39E-54	**VMDPAHEDKF**	**DFDPLDVTKIWPEDILPLQPVGRLVLNKNIDNFFNENEQIAFCPA**	**LVVPGIHYSD**
AtCAT3	283	5.19E-48	**TMDPADEDKF**	**DFDPLDVTKIWPEDILPLQPVGRLVLNRTIDNFFNETEQLAFNPG**	**LVVPGIYYSD**
OsCatA	283	5.19E-45	**VIDPEEEERF**	**DFDPLDDTKTWPEDEVPLRPVGRLVLNRNVDNFFNENEQLAFGPG**	**LVVPGIYYSD**
CsCAT3	274	2.29E-43	**TIDYDDQNNF**	**DFEPLDTTIEWPEDVIPLQPVGRLVLNKNIDNFFAENEMLAFSMS**	**LVPGIHYSDD**
CsCAT4	346	4.05E-37	**LIPEEDEFKF**	**DFDLLDPTKLIPEELVPVQLVGKMMLNRNPDNFFAENEQAAFHPG**	**HIVPGLDFTN**

CM-8

Name	Start	p-value		Sites	
PtCAT2	335	1.93E-65	**CPAIVVPGIY**	**YSDDKLLQTRIFSYADTQRHRLGPNYLQLPVNAPKCAHHNNHHEGFMNFM**	**HRDEEVNYFP**
PtCAT3	335	1.11E-64	**CPAIIVPGIY**	**YSDDKLLQTRIFSYADTQRHRLGPNYLQLPANAPKCAHHNNHHEGFMNFM**	**HRDEEVNYFP**
PtCAT1	335	1.11E-64	**CPAIVVPGVY**	**YSDDKLLQTRIFSYADTQRHRLGPNYLQLPANAPKCAHHNNHHEGFMNFM**	**HRDEEVNYFP**
AtCAT2	335	2.10E-64	**CPAIIVPGIH**	**YSDDKLLQTRVFSYADTQRHRLGPNYLQLPVNAPKCAHHNNHHEGFMNFM**	**HRDEEVNYFP**
AtCAT1	335	9.06E-64	**CPALVVPGIH**	**YSDDKLLQTRIFSYADSQRHRLGPNYLQLPVNAPKCAHHNNHHDGFMNFM**	**HRDEEVNYFP**
CsCAT2	301	1.33E-63	**CPAIIVPGIY**	**YSDDKLLQTRIFSYSDTQRHRLGPNYLQLPANAPKCAHHNNHHEGFMNFM**	**HRDEEVNYFP**
CsCAT1	334	2.99E-63	**CPAIIVPGIY**	**YSDDKLLQTRIFSYADTQRYRLGPNYLQLPVNAPKCAYHNNHHDGFMNFM**	**DRDEECTQPE**
AtCAT3	335	3.54E-63	**NPGLVVPGIY**	**YSDDKLLQCRIFAYGDTQRHRLGPNYLQLPVNAPKCAHHNNHHEGFMNFM**	**HRDEEINYYP**
OsCatB	335	7.11E-61	**CPAIIVPGIH**	**YSDDKLLQTRIFSYADTQRHRLGPNYLMLPVNAPKCAYHNNHHDGSMNFM**	**HRDEEVNYFP**
CsCAT3	325	8.97E-60	**FSMSLVPGIH**	**YSDDKMLQARSFAYADTQRHRLGPNYLQLPVNAPKCPHHNNHHEGFMNFM**	**HRDEEVNYFP**
OsCatC	335	1.68E-59	**CPGIIVPGIY**	**YSDDKLLQTRIFSYSDTQRHRLGPNYLLLPPNAPKCAHHNNHYDGFMNFM**	**HRDEEVDYFP**
OsCatA	335	3.35E-58	**GPGLVVPGIY**	**YSDDKMLQCRVFAYADTQRYRLGPNYLMLPVNAPKCAHHNNHYDGAMNFM**	**HRDEEVDYYP**

CM-9

Name	Start	p-value		Sites	
AtCAT2	385	3.84E-35	**NHHEGFMNFM**	**HRDEEVNYFPSRYDQVRHAEKYPTPPAVC**	**SGKRERCIIE**
CsCAT2	351	7.78E-35	**NHHEGFMNFM**	**HRDEEVNYFPSRYDPARHAERYPHPPAVC**	**TGKRERCVIQ**
PtCAT1	385	2.29E-34	**NHHEGFMNFM**	**HRDEEVNYFPSRYDPVRHAESFPIPPAVC**	**SGKREKCIIE**
CsCAT3	375	1.67E-32	**NHHEGFMNFM**	**HRDEEVNYFPSRYDPCRHAEKFPMPPNVL**	**SGKRERCVIP**
PtCAT2	385	3.33E-32	**NHHEGFMNFM**	**HRDEEVNYFPSRHDPSRHAERFPIPSAVC**	**SGRREKCIIE**
AtCAT1	385	3.73E-32	**NHHDGFMNFM**	**HRDEEVNYFPSRLDPVRHAEKYPTTPIVC**	**SGNREKCFIG**
OsCatB	385	5.68E-30	**NHHDGSMNFM**	**HRDEEVNYFPSRFDAARHAEKVPIPPRVL**	**TGCREKCVID**
PtCAT3	385	1.12E-29	**NHHEGFMNFM**	**HRDEEVNYFPSRFDPVRHAERYPNPSVIH**	**TGKREKCIIE**

OsCatC	385	4.90E-28	**NHYDGFMNFM**	**HRDEEVDYFPSRYDPAKHAPRYPIPSATL**	**TGRREKVVIA**
AtCAT3	385	1.32E-25	**NHHEGFMNFM**	**HRDEEINYYPSKFDPVRCAEKVPTPTNSY**	**TGIRTKCVIK**
OsCatA	385	2.86E-22	**NHYDGAMNFM**	**HRDEEVDYYPSRHAPLRHAPPTPITPRPV**	**VGRRQKATIH**
CsCAT1	429	1.12E-21	**MHIEQAQLLG**	**NLIFMINYFPSRIDPTRHAERYPQPSAVY**	**TGKRERCVIE**

CM-10

Name	Start	p-value		Sites	
PtCAT1	424	7.46E-65	**SGKREKCIIE**	**KENNFKQPGERYRSWAPDRQERFICRWVDALSDPRVTHEIRSIWISYWSQ**	**ADKSLGQKLA**
PtCAT2	424	6.25E-62	**SGRREKCIIE**	**KENNFKQPGERYRSWAPDRQERFVRRWVDALSEPRVTHEVRSIWISYWSQ**	**ADKSLGQKLA**
CsCAT2	390	1.11E-60	**TGKRERCVIQ**	**KENNFKEPGERYRSWTPDRQERFIRRWVDALSDPRVTHEIRSIWITYWSQ**	**ADRSVGQKLA**
CsCAT1	468	6.96E-60	**TGKRERCVIE**	**KENNFKQPGERYRSWPSDRQERFVGRWVDALSDPRVTHEIRNIWISYWSQ**	**ADKSLGHKLA**
AtCAT1	424	4.05E-59	**SGNREKCFIG**	**KENNFKQPGERYRSWDSDRQERFVKRFVEALSEPRVTHEIRSIWISYWSQ**	**ADKSLGQKLA**
OsCatC	424	1.44E-57	**TGRREKVVIA**	**KENNFKQPGERYRSWDPARQDRFIKRWIDALSDPRLTHEIRSIWLSYWSQ**	**ADRSLGQKLA**
PtCAT3	424	2.46E-57	**TGKREKCIIE**	**KENNFKQPGERYRSFSPDRKERFVRRWVEALSDPRVTYEIRSIWISYWSQ**	**ADKSLGQKLA**
AtCAT3	424	9.44E-56	**TGIRTKCVIK**	**KENNFKQAGDRYRSWAPDRQDRFVKRWVEILSEPRLTHEIRGIWISYWSQ**	**ADRSLGQKLA**
AtCAT2	424	9.44E-56	**SGKRERCIIE**	**KENNFKEPGERYRTFTPERQERFIQRWIDALSDPRITHEIRSIWISYWSQ**	**ADKSLGQKLA**
OsCatB	424	3.60E-53	**TGCREKCVID**	**KENNFQQAGERYRSFDPARQDRFLQRWVDALSDPRITHELRGIWISYWSQ**	**CDASLGQKLA**
CsCAT3	415	1.19E-51	**GKRERCVIPK**	**ENHNFKQAGDRYRSWAPDRQERFVRRFVEALSDPRVTHEVRNIWISYWSQ**	**ADRSLGQKIA**
OsCatA	424	1.95E-42	**VGRRQKATIH**	**KQNDFKQPGERYRSWAPDRQERFIRRFAGELAHPKVSPELRAIWVNYLSQ**	**CDESLGVKIA**
