# Supplementary material for: The catalase gene family in cucumber: genome-wide identification and organization
Source: Genet Mol Biol. 2016 Jul 25;39(3):408–15. doi: 10.1590/1678-4685-GMB-2015-0192 (PMC5004828; doi:10.1590/1678-4685-GMB-2015-0192)
Supplement: Supplementary file 3 [file 1415-4757-gmb-1678-4685-GMB-2015-0192-Suppl03.pdf]

Table S2. Sequence alignment between cucumber four *CsCAT* genes.

	CsCAT1	*CsCAT2*	*CsCAT3*	*CsCAT4*
CsCAT1	100	76	68	49
CsCAT2		100	70	40
CsCAT3			100	41
CsCAT4				100
