# Supplementary material for: The catalase gene family in cucumber: genome-wide identification and organization
Source: Genet Mol Biol. 2016 Jul 25;39(3):408–15. doi: 10.1590/1678-4685-GMB-2015-0192 (PMC5004828; doi:10.1590/1678-4685-GMB-2015-0192)
Supplement: Supplementary file 4 [file 1415-4757-gmb-1678-4685-GMB-2015-0192-Suppl01.pdf]

Figure S1. Multiple alignment of the deduced catalase amino acid sequences from cucumber. The three conserved catalytic amino acids (His, Asn and Tyr) are highlighted with a green background and the catalase proximal active site signature (FDRERIPERVVHAKGAGA) and proximal heme-ligand signature sequence (RLFSYNDTH) are *framed*. Carboxy terminal regions that have been implicated in importing targets into peroxisomes are *boxed* 1 to 3.

```
CsCAT2          ---------------------MD-----PYRHRPSSGYNTP-------------------
CsCAT1          ---------------------MY-----SHQ-RPSSAFDTP-------------------
CsCAT3          ---------------------MD-----PYKYRPSSAYNTP-------------------
CsCAT4          MSNNDKPHQSPIHGTEESQPGMDSLAPADGSHKPSPGPSAPGEQPTAPGSMKAPETENEK

CsCAT2          --------------FWTTNSGAPVWNNNSSLTVGPRGPILLEDYHLVEKLANFDRERIPE
CsCAT1          --------------FWTTNSGAPVWNNNSALTIGSRGPILLEDYHLVEKLANFDRERIPE
CsCAT3          --------------FCTTNSGAPVWNNT---------AVMSEDYQLIEKIATFTRERIPE
CsCAT4          LKSLDPHRKGGEGHALTTNQGVRIADDQNSLRAGTRGPTLLEDFILREKITHFDHERIPE

CsCAT2          R----------------------------------TPVIVRFSTVIHERGSPETLRDPRG
CsCAT1          RVVHARGASAKGFFEVTHDITHLTCADFLRAPGTQTPVIVRFSTVIHERGSPETLRDPRG
CsCAT3          RVVHARGASAKGFFEVTHDVSDLTCADFLRAPGVQTPVIVRFSTVIHERGSPETLRDPRG
CsCAT4          RIVHARGSAAHGYFQPYKSLKEITKADFLSDPNKITPVFVRFSTVQGGAGSADTVRDIRG

CsCAT2          FAVKFYTREGNFDLVGNNFPVFFIRDGMKFPDMVHALKPNPKSHIQEN----WRILDFFS
CsCAT1          FAVKFYTREGNFDLVGNNFPVFFVRDGMKFPDMVHALKPNPKSHIQEN----WRILDFFS
CsCAT3          FAVKFYTREGNFDIVGNNFPVFFVRDAMQFPDVIRAFKPNPKSHIQEP----WRILDFCS
CsCAT4          FATKFYTEEGIFDLVGNNTPVFFIQDAHKFPDFVHAVKPEPHWAIPQGQSAHDTFWDYVS

CsCAT2          HHPESLNMFTFLFDDIGIPQDYRHMDGSGVNTYTLINKAGKAHYVKFHWRPTCGVKSLLE
CsCAT1          HHPESLHMFTFLFDDLGIPQDYRHMDGSGVNTYTLINKEGKVHYVKFHWRPTCGVKTLLD
CsCAT3          YHPESLLSFAWFYDDVGIPINYRHMEGFGVQAYSLINKSGKARLVKFHWKPTCGVKSMLE
CsCAT4          LQPETLHNVMWAMSDRGIPRSYRTMEGFGIHTFRLINAEGKATFVRFHWKPVAGKASLVW

CsCAT2          EDAIRVGGSNHSHATQDLYDSIAAGNYPEWKLFIQTIDPDHEDRYDFDPLDVTKTWPEDI
CsCAT1          EVAIRVGGSNHSHATQDLYDSIAAGNYPEWELYIQTIDPDHEDKYDFDPLDVTKTWPEDI
CsCAT3          EEAIRIGGTNHSHATQDLYESIAAGNFPEWRLYIQTIDYDDQNNFDFEPLDTTIEWPEDV
CsCAT4          DEAQKLTGRDPDFHRRELWESIEAGDFPEYELGLQLIPEEDEFKFDFDLLDPTKLIPEEL

CsCAT2          LPLQPVGRMVLNKNIDNFFAENEQLAFCPAIIVPGIYYSDDKLLQTRIFSYSDTQRHRLG
CsCAT1          LPLQPVGRLVLNKNIDNFFAENEQLAFCPAIIVPGIYYSDDKLLQTRIFSYADTQRYRLG
CsCAT3          IPLQPVGRLVLNKNIDNFFAENEMLAFS-MSLVPGIHYSDDKMLQARSFAYADTQRHRLG
CsCAT4          VPVQLVGKMMLNRNPDNFFAENEQAAFHPGHIVPGLDFTNDPLLQGRLFSYSRLG----G

CsCAT2          PNYLQLPANAPKCAHHNNHHEGFMNFMHRDEE----------------------------
CsCAT1          PNYLQLPVNAPKCAYHNNHHDGFMNFMDRDEECTQPEFCACQHICWTDKYLLGMDTEVAM
CsCAT3          PNYLQLPVNAPKCPHHNNHHEGFMNFMHRDEE----------------------------
```

```
CsCAT4          PNFHELPINRPTCPYHNFQRDG-MHRQDIDTN----------------------------

CsCAT2          -----------------VNYFPSRYDPARHAERYPHP--------PAVCTGKRERCVIQK
CsCAT1          VVMHIEQAQLLGNLIFMINYFPSRIDPTRHAERYPQP--------SAVYTGKRERCVIEK
CsCAT3          -----------------VNYFPSRYDPCRHAEKFPMP--------PNVLSGKRERCVIPK
CsCAT4          ----------------PANYEPNSINDNWPRETPPGPKRGGFESYQERVDGTKIRERSPS

CsCAT2          EN-NFKEPGERYRSWTPDRQERFIRRWVDALSDPRVTHEIRSIWITYWSQADRSVGQKLA
CsCAT1          EN-NFKQPGERYRSWPSDRQERFVGRWVDALSDPRVTHEIRNIWISYWSQADKSLGHKLA
CsCAT3          ENHNFKQAGDRYRSWAPDRQERFVRRFVEALSDPRVTHEVRNIWISYWSQADRSLGQKIA
CsCAT4          FGEYYAQPRLFWNSQTPIEQQHIIGGFSFELS-KVVRTYIRERVVDHLAHIDIQLAQGVA
                                                                           3
CsCAT2          SHLNVRPSI---------------------------------------------------
CsCAT1          SHFNLKINT---------------------------------------------------
CsCAT3          SRMNVRPNI---------------------------------------------------
CsCAT4          NNLGITLTDEQCHAAPPKDVNGLKKDPSLSLYAVPGGTIKGRVVAILLNDKPRASDVLGI
                 1     2
CsCAT2          ------------------------------------------------------------
CsCAT1          ------------------------------------------------------------
CsCAT3          ------------------------------------------------------------
CsCAT4          MRALKTQGVHAKLLYSRMGEVTADDGSVLPIAATFAGAPSLTVDAVIMPCGDVESLLGNG

CsCAT2          ------------------------------------------------------------
CsCAT1          ------------------------------------------------------------
CsCAT3          ------------------------------------------------------------
CsCAT4          DAAYYLLEAYKHLKPIALAGDARKFKSLLKVPDQGEEGIVEGDNIDDAFMTRLFYLLAAH

CsCAT2          --------------
CsCAT1          --------------
CsCAT3          --------------
CsCAT4          RVWSRSSKIDQIPA
```
